# Esterases From *Bifidobacteria* Exhibit the Conversion of Albiflorin in Gut Microbiota

**DOI:** 10.3389/fmicb.2022.880118

**Published:** 2022-04-06

**Authors:** Ran Peng, Pei Han, Jie Fu, Zheng-Wei Zhang, Shu-Rong Ma, Li-Bin Pan, Yuan-Yuan Xia, Hang Yu, Hui Xu, Chang-Xiao Liu, Yan Wang

**Affiliations:** ^1^State Key Laboratory of Bioactive Substance and Function of Natural Medicines, Institute of Materia Medica, Chinese Academy of Medical Sciences and Peking Union Medical College, Beijing, China; ^2^Tianjin Institute of Pharmaceutical Research, Research Unit for Drug Metabolism, Chinese Academy of Medical Sciences, Tianjin, China

**Keywords:** gut microbiota, genome mining, esterase structure, metabolism, *Bifidobacteria*, albiflorin, benzoic acid

## Abstract

*Bifidobacteria* is an important microbe that inhabits the human gut. It is capable of metabolizing complex compounds in the human diet. Albiflorin, an antidepressant natural product from *Radix Paeoniae Alba* in China, is difficult to absorb after oral administration, and its metabolism has been proven to be closely related to the gut microbiota. In this study, we demonstrated *in vitro* that several *Bifidobacteria* species were able to convert albiflorin to benzoic acid, and four esterases (B2, B3, B4, and BL) from *Bifidobacterium breve* and *Bifidobacterium longum* were found through genome mining and modeled by SWISS-MODEL. B2 and B3 presented the strongest albiflorin metabolism ability. The optimal conditions, including temperature, buffer, and pH, for the conversion of albiflorin by the four esterases were investigated. Furthermore, the effect of esterase on the metabolism of albiflorin *in vivo* was confirmed by transplanting bacteria containing esterase B2. This study demonstrated the vital role of esterases from *Bifidobacteria* in the metabolism of natural compounds containing ester bonds, which could contribute to the development of new enzymes, microbial evolution, and probiotic adjuvant compounds for treatment.

## Introduction

The human gut microbiota is mainly composed of anaerobic bacteria, and the number of cells per gram of intestinal contents exceeds 10^11^ (Sender et al., 2016). The dense gut microbiota greatly affects the host’s metabolic capacity, nutritional status, and immune system development (Nicholson et al., 2012). In adults, intestinal bacteria mainly comprise species from Firmicutes, Bacteroides, Actinobacteria, and Proteobacteria. Actinomycetes are mainly represented by species of the genus *Bifidobacterium*, accounting for 2–10% of adult intestinal bacteria (Turroni et al., 2008; Arumugam et al., 2011). Nowadays, gut microbiota deviations are linked with many diseases including obesity, type 2 diabetes, hepatic steatosis, intestinal bowel diseases (IBDs), and several types of cancer (de Vos et al., 2022). The gut microbiota has both direct and indirect effects on drug and xenobiotic metabolisms, and this can have consequences for both efficacy and toxicity (Wilson and Nicholson, 2016). *Bifidobacteria* are some of the most important probiotics in gut microbiota (Rossi and Amaretti, 2010). They play a beneficial role *via* multiple mechanisms, such as immune stimulation, anticancer activity, inhibition of pathogen growth, production of vitamins and amino acids, reduction of cholesterol, alleviation of the symptoms of irritable bowel syndrome, treatment of mood disorders, and bioconversion of a variety of natural compounds into bioactive compounds (Whorwell et al., 2006; Fanning et al., 2012; Ventura et al., 2014; Zanotti et al., 2015). Therefore, research on the relationship between humans and *Bifidobacteria*, including its beneficial effects on human health and its symbiosis mechanism, has received extensive attention. As important probiotics, *Bifidobacteria* species possess abundant enzymes that are of great significance. Recent studies have shown that the esterase of *Bifidobacterium longum* can metabolize hydroxycinnamic acid from food (Kelly et al., 2018), and the sialidase of *Bifidobacterium bifidum* can catalyze the removal of terminal sialic acids from various complex carbohydrates (Ashida et al., 2018). The esterases in *Bifidobacteria* can metabolize chlorogenic acid in food (Raimondi et al., 2015). However, little is known about the esterases of *Bifidobacteria* and drug metabolism.

As the main component of Xiaoyao Wan, a common Chinese patent medicine prescribed for the treatment of depression-like disorders, albiflorin has poor bioavailability (5.4%) and can hardly cross the blood–brain barrier. The level of albiflorin in the blood and brain is considerably low after oral administration (Huang et al., 2014). Previous research by our group found that *Bifidobacteria* in intestinal bacteria are closely related to the metabolism of albiflorin. The characteristic metabolite is benzoic acid (BA). The esterase in *Bifidobacteria* may be one of the key metabolic enzymes in the metabolic process (Zhao et al., 2018). Similarly, the esterase of *Bifidobacteria* is involved in the conversion of albiflorin, which is also the main component of Xiaoyao Wan (Yu et al., 2019). Esterases and lipases are two major hydrolases that can cleave ester bonds. Both of them have an α/β-hydrolase fold and contain a consensus sequence (Gly-X-Ser-X-Gly) adjacent to the catalytic triad Ser-Asp-His (Kroon et al., 2000; Bornscheuer, 2002). In contrast to lipases, esterases generally obey classical Michaelis-Menten kinetics and hydrolyze compounds that have less than six carbons (Arpigny and Jaeger, 1999; Bornscheuer, 2002).

This study aimed to explore the ability of probiotic *Bifidobacteria* species to hydrolyze albiflorin into benzoic acid and to identify the enzymes involved in this reaction. Hydrolysis seems to be a characteristic phenomenon of *Bifidobacteria*. Four *Bifidobacteria* strains found in the human intestine were chosen for the conversion of albiflorin. We detected the activity of esterases in *Bifidobacterium breve* and *B. longum*, which can hydrolyze albiflorin. By genome mining and modeling with SWISS-MODEL, four esterases were found, and they all have the core domain of esterase, but the surrounding regions are very different. The functional characterization of the four esterases, which were expressed in *Escherichia coli*, was performed and compared under different conditions. Further, we proved the important role of esterase B2 in affecting the metabolism of albiflorin and improving the concentration of benzoic acid in plasma by pharmacokinetic study *in vivo* with bacteria transplantation. This demonstrated the possible role of *Bifidobacteria* in albiflorin metabolism, which revealed new prospects for the development of novel enzyme preparations and probiotics specifically designed for the enhancement of the bioconversion of traditional Chinese medicinal (TCM) chemicals into biologically active compounds.

## Materials and Methods

### Chemicals, Bacterial Strains, and Culture Conditions

Albiflorin, benzoic acid, propranolol, anaerobic medium, and MRS broth medium were all purchased from Solarbio (Beijing, China). The purity of the compounds was higher than 98% (HPLC). Albiflorin and benzoic acid were dissolved to prepare a 0.5 mmol/L stock solution in water. HPLC-grade ammonia, acetonitrile, and methanol were obtained from Fisher Scientific (Fair Lawn, NJ, United States).

The bacterial strains and plasmids employed in this study are listed in Supplementary Table S1. Four *Bifidobacteria* strains (*B. breve* ATCC15700, *B. longum* ATCC15697, *Bifidobacterium animalis* ATCC27673, and *Bifidobacterium adolescentis* ATCC15703) were provided by the ATCC Biological Resource Center. The *Bifidobacteria* strains were cultured in MRS medium at 37°C under anaerobic conditions in N_2_ gas. The *E. coli* strains DH5α and BL21 (DE3) were used to clone the pET-28a plasmid and express the esterase protein, respectively. The *E. coli* strain was grown in Luria-Broth (LB) medium (10 g/L trypsin, 5 g/L yeast extract, and 5 g/L NaCl, pH 7.2) at 37°C. If necessary, 10–15 μg/ml kanamycin (km) was added to the medium.

The concentrations of albiflorin and benzoic acid were determined using an HPLC-MS/MS 8050 system from Shimadzu Corporation (Kyoto, Japan). The substance to be tested was separated in liquid phase with an Alltima C_18_ column (100 mm × 2.1 mm × 5 μm, Grace, England). The gradient elution started with 80% mobile phase A (water) and decreased to 50% mobile phase A over 5 min at a flow rate of 0.4 ml/min. Then, it was maintained at 50% for 2 min and returned to 80% mobile phase A over 1 min, where it was maintained for 10 min. Mobile phase B consisted of methanol. The column temperature and autosampler temperature were set at 30°C and 4°C, respectively. The mass spectrometer was run in multiple reaction monitoring (MRM) mode: 503.00 → 341.05 (*m/z*) for albiflorin (+), 121.10 → 77.10 (*m/z*) for benzoic acid (−), and 260.20 → 116.10 (*m/z*) for propranolol (−). The sample processing method was as follows: 100 μl of sample was added to 300 μl of methanol containing 50 ng/ml of internal standard (propranolol). Then, the mixture was vortexed for 30 s and centrifuged at 12,000 rpm for 10 min. The supernatant was prepared for quantitative analysis by LC-MS/MS, and the injection volume was 1 μl.

### Animals

Sprague-Dawley (SD) male rats weighing 200 ± 20 g and ICR mice weighting 18 ± 22 g were provided by the Institute of Laboratory Animal Science, Chinese Academy of Medical Sciences (Beijing, China). The animals were placed in a cage with free access to chow and water. The temperature of the cage was kept at approximately 22°C with 50% humidity and a 12-h day/night cycle. Rats were fasted for 12 h before the experiment but had free access to water. This study was conducted in accordance with institutional and ethics guidelines approved by the Laboratory Institutional Animal Care and Use Committee of the Chinese Academy of Medical Sciences and Peking Union Medical College.

### Bioconversion of Albiflorin With Rat Intestinal Bacteria and *Bifidobacteria* Strains

We obtained the colon contents from six SD rats and transferred 5 g of the collected sample into 100 ml anaerobic medium. Then, the mixture was preincubated under anaerobic conditions (N_2_ atmosphere) at 37°C for 1 h. After preincubation, 10 μl of albiflorin was transferred into 990 μl of the culture. The cultures were inactivated by high temperature and high pressure at 121°C as a negative control. The final concentrations of albiflorin in the incubation system were 0.05 mM and 0.5 mM. The cultures were incubated at 37°C for 0, 12, or 24 h. The method was performed in accordance with the previously described procedure (He et al., 2017).

Albiflorin and its metabolite were analyzed by an Alltima C_18_ column (100 mm × 2.1 mm × 5 μm, Grace, England) with an LC/MS^n^-IT-TOF system (Shimadzu Corporation, Kyoto, Japan) in both positive and negative modes. Mass spectra were acquired in the range of *m/z* 100–1,000 for MS^1^. The MS^n^ data were collected in automatic mode. An elution gradient was employed at a flow rate of 0.4 ml/min, with water as mobile phase A and methanol as mobile phase B, by using the following program: 0.10 min (90% A and 10% B), 3.00 min (60% A and 40% B), 7.00 min (40% A and 60% B), 9.00 min (10% A and 90% B), 9.01 min (90% A and 10% B), and 12.00 min (controller stop).

Four strains of *Bifidobacteria*, *B. breve* ATCC15700, *B. longum* ATCC15697, *B. animalis* ATCC27673, and *B. adolescentis* ATCC15703 were cultured to convert albiflorin *in vitro*. After resuscitation, the strains were cultured overnight in MRS medium at 37°C under anaerobic conditions (N_2_ atmosphere), and the bacterial concentration was uniformly maintained at 3 × 10^6^ cells/ml. The cultures were incubated at 37°C for 0, 12, and 24 h, and the final concentration of albiflorin in the incubation system was 0.05 mM. The contents of albiflorin and its metabolite benzoic acid were determined and analyzed by LC-MS/MS.

### Bioinformatics Analyses

The NCBI database^1^ was used to search the genomes of *B. breve* and *B. longum* for genes labeled “carboxylesterase” and “esterase.” By searching for genes annotated as “carboxylesterase” in the genome of *B. breve* ATCC15700 (NZ_AP012324.1), we found three carboxylesterase genes, NZ_AP012324.1: 1013236–1014057, NZ_AP012324.1: 506173–507018, and NZ_AP012324.1: 506173–507018. The amino acid sequences can be accessed as WP_003829196.1 (B2, 273 aa), WP_003828396.1 (B3, 281 aa), and WP_003828023.1 (B4, 319 aa). By searching for the gene annotated as “esterase” in the genome of *B. longum* ATCC15697 (NC_011593.1), we found an esterase gene, NC_011593.1: c2783438-2782680, and its protein sequence can be found in KAB6720564.1 (BL, 252 aa). Detailed information on each protein was obtained from the NCBI database, as shown in Table 1.

**Table 1:** The information of esterases in *Bifidobacteria* strains.

Name	NCBI reference sequence	Source	Number of amino acids	Molecular weight	Theoretical pI	Superfamily	Accession	Definition
B2	WP_003829196.1	*Bifidobacterium breve* ATCC 15700	273	30,219.38	5.19	MhpC	cl33968	Alpha/beta hydrolase
B3	WP_003828396.1	*Bifidobacterium breve* ATCC 15700	281	30,261.08	4.50	protocat_pcaD	cl31213	Alpha/beta hydrolase
B4	WP_003828023.1	*Bifidobacterium breve* ATCC 15700	319	35,264.78	5.22	MhpC	cl33968	Alpha/beta hydrolase
BL	KAB6720564.1	*Bifidobacterium longum* ATCC 15697	252	27,695.84	4.79	Abhydrolase	cl21494	Esterase

3-D structural modeling of the esterase was conducted with SWISS-MODEL,^2^ which was the first fully automated protein homology modeling server (Guex and Peitsch, 1997; Schwede et al., 2003; Bordoli et al., 2009). The SWISS-MODEL workspace can be freely accessed at http://swissmodel.expasy.org/workspace/. We used the automatic modeling mode and applied the protein sequences of four esterases, which were available in GenBank. The data obtained from the homology model were visualized using DeepView ver. 4.0.1 (The Swiss Institute of Bioinformatics).

### Plasmids Construction for Heterologous Expression of the Esterases From *Bifidobacterium breve* and *Bifidobacterium longum*

To obtain the genomic DNA of *B. breve* and *B. longum*, extraction was carried out according to the instructions of the bacterial genome extraction kit (Tiangen, China). Using the genomic DNA of *B. breve* and *B. longum* as templates, Primer Premier 5.0 was used to design primers based on the four esterase genes (*b2*, *b3*, *b4*, and *bl*) and the vector pET-28a. All of the primers (b2-F, b2-R, b3-F, b3-R, b4-F, b4-R, bl-F, bl-R, pET28a-F, and pET28a-R) are listed in Supplementary Table S2. By using the genomic DNA of *B. breve* and *B. longum* as templates, the four esterase genes were amplified by PCR and then purified along with the PCR-amplified vector DNA using the Gel Recovery Purification Kit (Tiangen, China). A Gibson Assembly Kit (NEB, United States) was used to insert the four esterase DNA fragments into the vector pET-28a to construct the recombinant vectors. *Escherichia coli* DH5α cells were transformed by the heat shock method and used for plasmid propagation to obtain the recombinant plasmids pET-28a-*b2*, pET-28a-*b3*, pET-28a-*b4,* and pET-28a-*bl*. All fragments were validated by Sanger 3730 sequencing.

### Expression of *b2, b3, b4,* and *bl* in *Escherichia coli* and Functional Verification

The recombinant plasmids pET-28a-*b2*, pET-28a-*b3*, pET-28a-*b4*, and pET-28a-*bl* were transformed into *E. coli* BL21 (DE3) cells. Cells were grown in liquid LB media supplemented with kanamycin (40 μg/ml). Cultures were maintained at 37°C until the OD_600 nm_ reached 0.6. Then, the temperature was lowered to 16°C, and protein expression was induced with 0.1 mM isopropyl β-D-1-thiogalactopyranoside (IPTG) for 20 h. The bacterial cells were harvested by centrifugation (12,000 × *g*, 30 min, 4°C), concentrated 10-fold, and suspended in approximately 10 ml of lysis buffer (10 mM PBS buffer or 25 mM Tris-HCl buffer). After ultrasonic lysis, the supernatant and precipitate were prepared for SDS-PAGE analysis, with the empty pET-28a plasmid in *E. coli* BL21 cells as a control. The B2 and B3 proteins were purified using the PrepEase His-tag protein purification kit (Takara, Japan).

*Escherichia coli* cells with B2, B3, B4, and BL and the cell extract supernatant were used for the bioconversion of albiflorin to characterize B2, B3, B4, and BL. *Escherichia coli* BL21 (DE3) transformed with the empty pET-28a vector was used as a negative control. The final concentration of albiflorin in the incubation system was 0.05 mM. The cultures were incubated at 37°C for 0, 12, and 24 h. Albiflorin and its metabolite benzoic acid were detected by LC-MS/MS. The enzymatic reaction conditions were as follows: temperatures, 30°C and 37°C; buffers, PBS buffer and Tris-HCl buffer; and pH, 6.0, 7.0, and 8.0. The LC-MS/MS conditions refer to the above experimental methods.

### Microbiota Transplantation and Pharmacokinetic Study *in vivo*

Three groups of ICR mice (*n* = 6/group) were orally administered *E. coli* (1 × 10^10^ CFU), the *E. coli* with esterase B2 (1 × 10^10^ CFU), and *B. breve* (1 × 10^10^ CFU) once a day for 3 days, respectively. Another six ICR mice were orally administered an equivalent volume of saline. Pharmacokinetic evaluations were performed 3 days after the final administration.

Before the oral administration of a single dose of albiflorin (7 mg/kg), the four groups of mice were fasted overnight with free access to water. Blood samples were collected before and at 5, 10, 20, 30, 45, 60, 90, 120, 180, 240, 360, and 480 min after drug treatment.

### Statistical Analysis

Statistical analyses were performed with GraphPad Prism Version 5 (GraphPad Software, CA, United States) using two-way ANOVA and Student’s *t*-test. Data are expressed as the mean ± standard deviation, and *p* values less than 0.05 were considered statistically significant.

## Results

### Conversion of Albiflorin Into Benzoic Acid in Gut Microbiota

**Figure 1:**
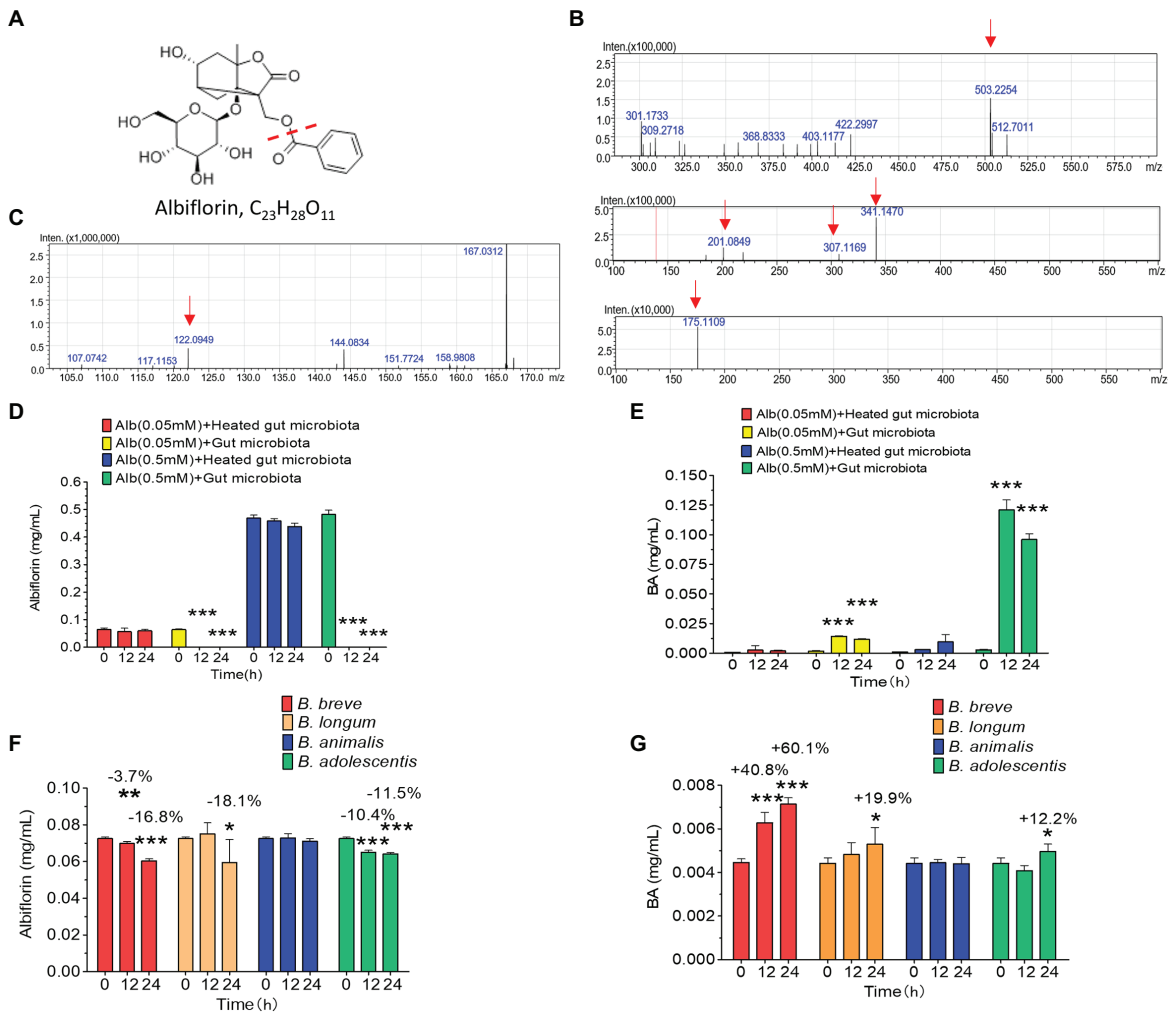
The intestinal microbes of rats converted albiflorin into benzoic acid. **(A)** Schematic diagram of the structure of albiflorin and benzoic acid. **(B)** High-resolution mass spectrum of albiflorin. **(C)** High-resolution mass spectrum of benzoic acid. **(D)** Conversion of albiflorin by the intestinal microbes of rats. **(E)** Benzoic acid (BA) generated by the intestinal microbes of rats. **(F)** Conversion of albiflorin in the four *Bifidobacteria* strains. **(G)** Benzoic acid (BA) generated by the four *Bifidobacteria* strains. (Student’s *t*-test, ^*^p < 0.05, ^**^p < 0.01, and ^***^p < 0.001, data are expressed as the mean ± SD).

Intestinal bacteria from SD rats were collected for anaerobic incubation in the presence of albiflorin, and the concentrations of albiflorin in the culture solution were 0.05 and 0.5 mM, respectively, with the inactivated intestinal bacteria culture functioning as a negative control. The structure of albiflorin (Figure 1A) includes a benzoyl group, which might be hydrolyzed by esterases present in gut microbiota. LC/MS^n^-IT-TOF was used for identifying albiflorin and BA. The mass spectrum of albiflorin is shown in Figure 1B. The *m/z* of the [M + Na]^+^ peak was 503, the *m/z* of the secondary fragments were 201, 307, and 341, and the tertiary ion fragment had an *m/z* of 175. The mass spectrum of benzoic acid is shown in Figure 1C, and the [M + H]^+^ peak of benzoic acid had an *m/z* of 122. Moreover, different concentrations of albiflorin (0.05 and 0.5 mM) were cultured with the intestinal bacterial culture and inactivated intestinal bacterial culture at 37°C for 12 and 24 h, and the concentrations of albiflorin and benzoic acid were determined after incubation. As shown in Figures 1D,E, regardless of the concentration, the albiflorin cultured with the intestinal bacteria solution was completely converted after 12 h, and the metabolite benzoic acid was produced. However, albiflorin cultured with the inactivated intestinal bacteria solution was not converted into benzoic acid. The results indicated that the intestinal bacteria of SD rats were able to metabolize albiflorin and convert it into benzoic acid.

### Bioconversion of Albiflorin by Four *Bifidobacteria* Strains

The capability of four common *Bifidobacteria* strains (*B. breve*, *B. longum*, *B. animalis*, and *B. adolescentis*) found among gut microbes to metabolize albiflorin under anaerobic conditions over a period of 24 h was examined. The cells were maintained at a consistent concentration with 3 × 10^6^ cells/ml in the presence of 0.05 mM albiflorin. Samples were removed at 0, 12, and 24 h for LC-MS/MS detection. As shown in Figures 1F,G, *B. breve*, *B. longum*, and *B. adolescentis* showed significant metabolism of albiflorin at 12 and 24 h, while *B. longum* had the strongest ability to metabolize albiflorin and resulted in a high conversion rate at 24 h of 18.1%, followed by the rates of *B. breve* (16.8%) and *B. adolescentis* (1.5%). Accordingly, benzoic acid was produced differently by the tested *Bifidobacteria* strains, resulting in 7.14 μg/ml for *B. breve*, 5.29 μg/ml for *B. longum*, and 4.96 μg/ml for *B. adolescentis*. According to the above results, *B. breve* and *B. longum* have a strong albiflorin metabolism ability, and we propose that both play an important role in albiflorin metabolism in the gut microbiota.

### *In silico* Search of *Bifidobacterium* Esterases

By searching for genes annotated as “carboxylesterase” in the genome of *B. breve* ATCC15700 (NZ_AP012324.1), three carboxylesterase genes were found, including NZ_AP012324.1: 1013236–1014057, NZ_AP012324.1: 506173–507018, and NZ_AP012324.1: 506173–507018. The corresponding amino acid sequences are WP_003829196.1 (B2, 273 aa), WP_003828396.1 (B3, 281 aa), and WP_003828023.1 (B4, 319 aa). By searching for the genes annotated as “esterase” in the genome of *B. longum* ATCC15697 (NC_011593.1), an esterase gene was found, which was named NC_011593.1:c2783438-2,782,680, and its protein identifier is KAB6720564.1 (BL, 252 aa). Detailed information on each protein was obtained from the NCBI database, and the ProtParam tool by ExPASy^3^ was used to predict the size and isoelectric point of each protein. As shown in Table 1, the predicted molecular weight of B2 was 30,219.38 Da, and the isoelectric point was 5.19. The values of the molecular weight and isoelectric point of the other proteins were as follows: B3: 30,261.08 Da, 4.50; B4: 35,264.78 Da, 5.22.; and BL: 27,695.84 Da, 4.79.

We found that the four enzymes, B2, B3, B4, and BL, belong to three superfamilies. As shown in Table 1, B2 and B4 belong to the MhpC superfamily, which is annotated as “Pimeloyl-ACP methyl ester carboxylesterase.” B3 belongs to the protocat_pcaD superfamily, which is annotated as “3-oxoadipate enol-lactonase. Note that the substrates are 3-oxoadipate enol-lactone, 2-oxo-2,3-dihydrofuran-5-acetate, 4,5-dihydro-5-oxofuran-2-acetate, and 5-oxo-4,5-dihydrofuran-2-acetate.” BL belongs to the abhydrolase superfamily, which is annotated as “alpha/beta hydrolases. A functionally diverse superfamily containing proteases, lipases, peroxidases, esterases, epoxide hydrolases and dehalogenases.”

**Figure 2:**
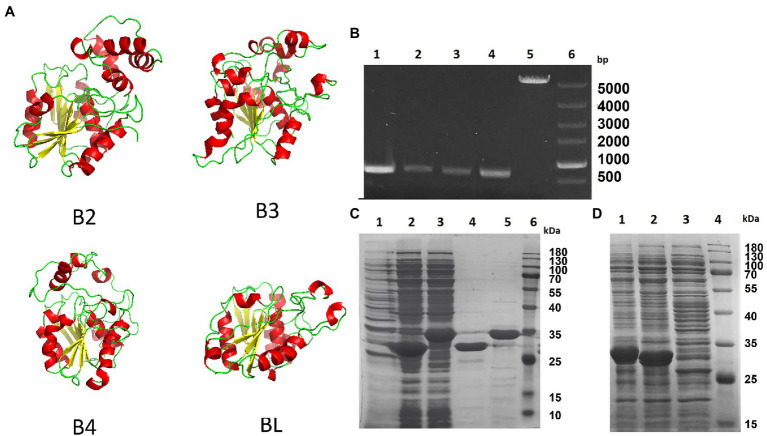
Homology model and expression of four *Bifidobacterium* esterases. **(A)** Homology model of the four esterases, B2, B3, B4, and BL, generated by SWISS-MODEL. **(B)** Agarose gel electrophoresis image showing the PCR-amplified products of four esterase genes from *Bifidobacterium breve* and *Bifidobacterium longum*. Lane 1, *bb2*; lane 2, *bb3*; lane 3, *bb4*; lane 4, *bl*; lane 5, pET-28a; and lane 6, supercoiled DNA marker. **(C)** SDS-PAGE analysis of two proteins, B2 and B3, from *B. breve* after being expressed in *Escherichia coli* BL21. Lane 1, total protein from the lysate of *E. coli* transformed with pET-28a; lane 2, total protein of *E. coli* lysate containing esterase B2; lane 3, total protein of *E. coli* lysate containing esterase B3; lane 4, esterase B2 after purification; lane 5, esterase B3 after purification; and lane 6, prestained protein molecular weight marker. **(D)** SDS-PAGE analysis of two esterases, B4 and BL, from *B. breve* and *B. longum* in *E. coli* BL21. Lane 1, total protein of *E. coli* lysate containing esterase B4; lane 2, total protein of *E. coli* lysate containing esterase BL; lane 3, total protein of *E. coli* lysate transformed with pET-28a; and lane 4, prestained protein molecular weight marker.

We modeled the four esterases, B2, B3, B4, and BL, by using SWISS-MODEL. As shown in Figure 2A, they all have the core domain of an esterase, but the surrounding regions are very different. The modeled structure showed that their core structures are similar and that they may all be esterases. These enzymes are composed of highly similar α/β-hydrolase folds and different helix domains which suggested that they have different cleavage patterns. They are esterases rather than lipases as they elicit a preference for smaller carbon backbone substrates less than six carbons. It has previously been reported that the bifidobacterial esterase from *B. animalis* subsp. *lactis* WC 0432 exhibits hydrolytic activity against chlorogenic acid (Raimondi et al., 2015).

### Expression of *Bifidobacterium* Esterases in *Escherichia coli*

To investigate the suitability of *E. coli* BL21 (DE3) for *Bifidobacterium* esterase expression, we first verified the absence of homologous genes in its genome by analyzing its genome, and the *E. coli* cells with the empty vector pET-28a were incapable of metabolizing any albiflorin (data not shown). Several primer pairs designed based on the B2, B3, B4, and BL coding sequences of *B. breve* ATCC15700 and *B. longum* ATCC15697 were used to clone the *four genes* (the primer sequences are shown in Supplementary Table S1). The four PCR-amplified esterase DNA fragments (as shown in Figure 2B) were recombined into the vector pET-28a by Gibson assembly. DH5α *E. coli* were used to clone the esterase coding sequences of *B. breve* and *B. longum*, and four plasmids, pET-28a-*b2*, pET-28a-*b3*, pET-28a-*b4*, and pET-28a-*bl*, were obtained. *Escherichia coli* BL21 (DE3) was transformed with the above expression vectors bearing four esterase genes. SDS-PAGE showed that the expression of the four esterases occurred at high levels in IPTG-induced recombinant cells (Figures 2C,D).

### Esterase Activity Characterization *in vitro*

**Figure 3:**
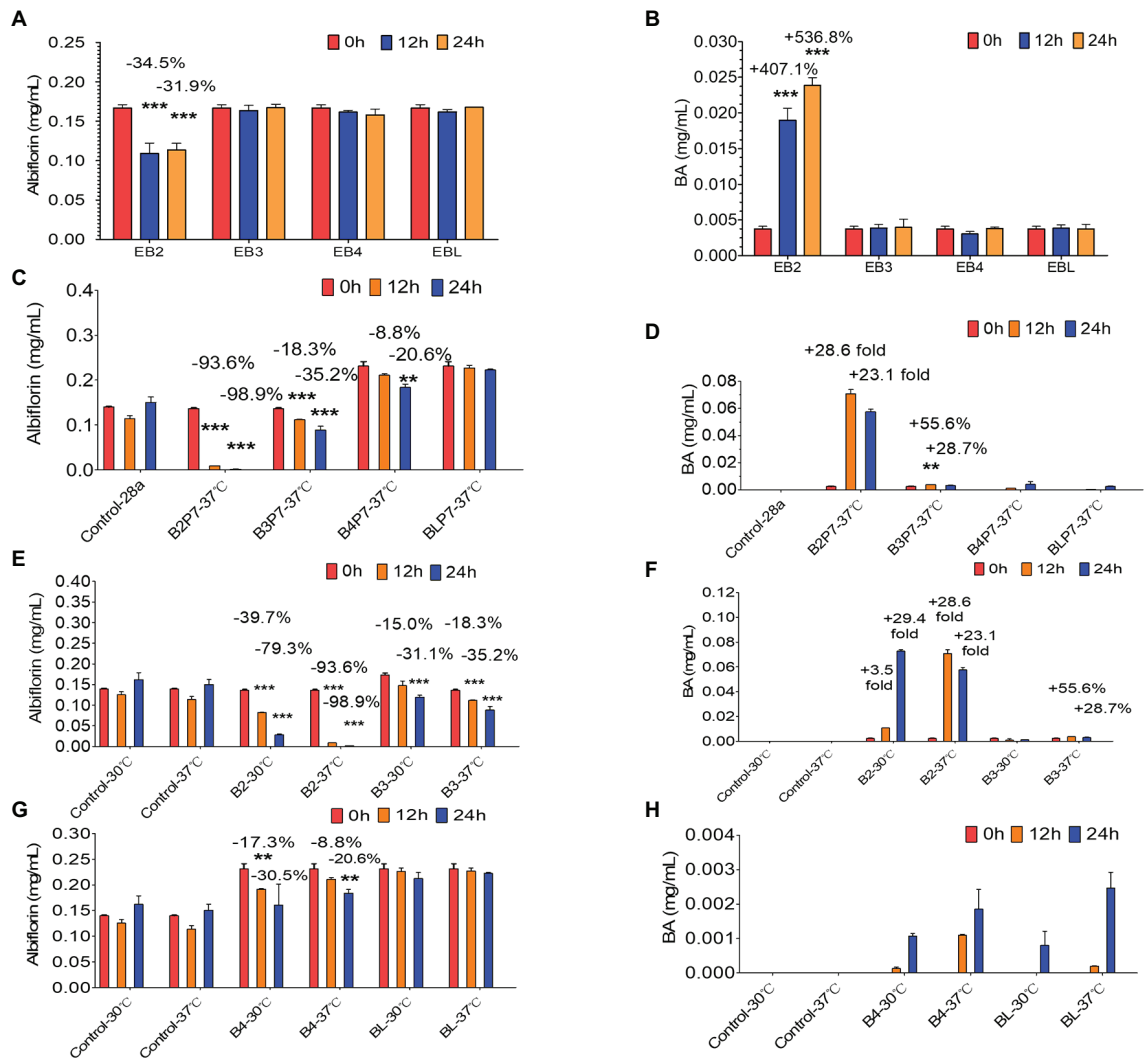
Albiflorin metabolism by four esterases (B2, B3, B4, and BL). **(A)** Conversion of albiflorin in *Escherichia coli* stains (EB2, EB3, EB4, and EBL). **(B)** Benzoic acid (BA) generated by the *E. coli* strains (EB2, EB3, EB4, and EBL). **(C)** Conversion of albiflorin by four esterases. **(D)** Benzoic acid (BA) generated by four esterases. **(E)** Conversion of albiflorin by B2 and B3 at 30°C or 37°C. **(F)** Benzoic acid (BA) generated by B2 and B3 at 30°C or 37°C. **(G)** Conversion of albiflorin by B4 and BL at 30°C or 37°C. **(H)** Benzoic acid (BA) generated by B4 and BL at 30°C or 37°C (Student’s *t*-test, ^**^p < 0.01 and ^***^p < 0.001, data are expressed as the mean ± SD).

Based on the above results, four *E. coli* strains (EB2, EB3, EB4, and EBL) expressing the four esterases were obtained. After being cultured overnight with IPTG, the four *E. coli* strains were individually used to investigate albiflorin metabolism at 37°C for 24 h. The cells of each strain were controlled at a consistent concentration of OD_600 nm_ = 2.0 in the presence of 0.05 mM albiflorin. Samples were removed at 0, 12, and 24 h for LC-MS/MS analysis. As shown in Figures 3A,B, *E. coli* EB2 had a significant effect on albiflorin metabolism, the level of which was reduced by 31.9% at 24 h compared with that at 0 h. Moreover, the production of benzoic acid at 24 h was increased by 5.37-fold compared with that at 0 h. The concentration of benzoic acid reached 23.9 μg/ml. However, EB3, EB4, and EBL had no effect on albiflorin metabolism, and no significant increase was detected in the production of benzoic acid. This result showed the metabolic ability of the four esterases expressed by *E. coli* cells in suspension culture, and the *E. coli* strain expressing esterase B2 showed the greatest potential for albiflorin metabolism.

Furthermore, the *E. coli* cells expressing the four esterases were collected and resuspended in PBS buffer (pH = 7) and sonicated to obtain the B2, B3, B4, and BL proteins. The supernatants were mixed with 0.05 mM albiflorin and incubated at 37°C. Samples were taken at 0, 12, and 24 h for the detection of albiflorin and its metabolite benzoic acid. As shown in Figure 3C, after 24 h of reaction, 98.9% of albiflorin was converted by B2, 35.2% by B3, and 20.6% by B4, but the conversion by BL was not significant. The production of benzoic acid is shown in Figure 3D. The concentrations of benzoic acid produced by B2 and B3 were 70.8 and 3.7 μg/ml, respectively, but the concentrations produced by B4 and BL were not obvious compared with those at 0 h. The results indicated that the B2 and B3 proteins have a strong ability to metabolize albiflorin, and B2 has the strongest conversion effect, followed by B3 and B4. Similarly, the B2 reaction system produced the most benzoic acid, followed by the B3 and B4 reaction systems, which was consistent with the above results.

### Effects of Esterases on Albiflorin Metabolism Under Different Conditions

Furthermore, the effects of different temperatures, buffers, and pH on the ability of the four enzymes to convert albiflorin were investigated. First, as shown in Figures 3E,F, after 24 h of reaction at 37°C, B2 and B3 converted 98.9 and 35.2% of albiflorin, respectively, which was greater than that observed at 30°C (79.3 and 31.1%). The production of benzoic acid at 37°C was also greater than that at 30°C. The conversion capability of B4 and BL are shown in Figures 3G,H. B4 converted 30.5% albiflorin at 30°C in 24 h, and the percentage at 37°C was 20.6%. However, there was no significant difference for BL between 30°C and 37°C in terms of albiflorin metabolism. Both temperatures led to the production of very little benzoic acid under the reaction conditions.

**Figure 4:**
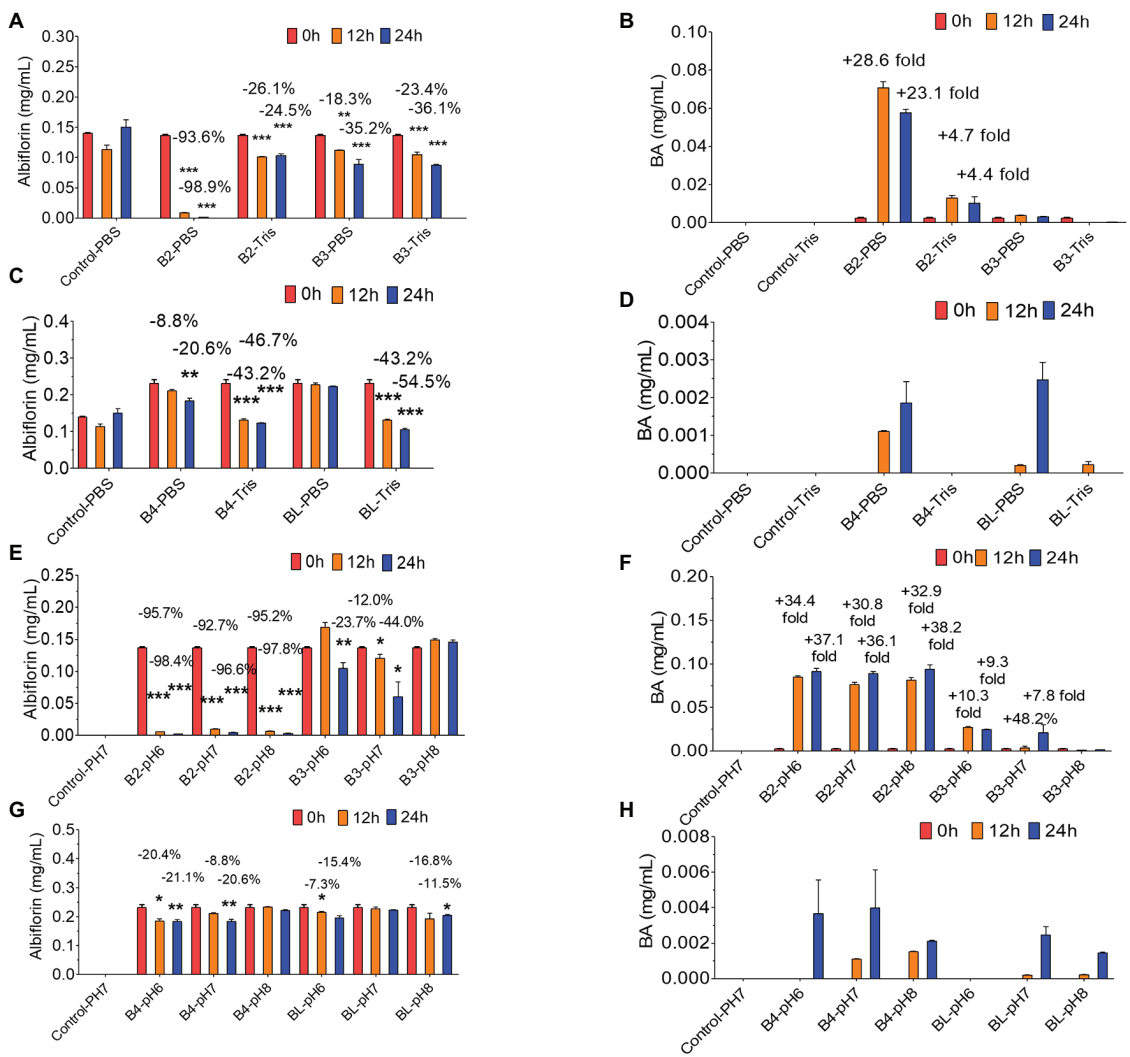
Albiflorin metabolism by four esterases (B2, B3, B4, and BL) under different conditions. **(A)** Conversion of albiflorin by B2 and B3 in PBS and Tris-HCl buffer. **(B)** Benzoic acid (BA) generated by B2 and B3 in PBS and Tris-HCl buffer. **(C)** Conversion of albiflorin by B4 and BL in PBS and Tris-HCl buffer. **(D)** Benzoic acid (BA) generated by B4 and BL in PBS and Tris-HCl buffer. **(E)** Conversion of albiflorin by B2 and B3 at pH 7.0, 8.0, and 9.0. **(F)** Benzoic acid (BA) generated by B2 and B3 at pH 7.0, 8.0, and 9.0. **(G)** Conversion of albiflorin by B4 and BL at pH 7.0, 8.0, and 9.0. **(H)** Benzoic acid (BA) generated by B4 and BL at pH 7.0, 8.0, and 9.0 (Student’s *t*-test, ^*^p < 0.05, ^**^p < 0.01, and ^***^p < 0.001, data are expressed as the mean ± SD).

Then, the effect of the reaction conditions of the PBS buffer and Tris-HCl buffer on the conversion of albiflorin by the four enzymes was investigated. As shown in Figures 4A,B, under the reaction conditions of the PBS buffer solution, after 24 h of reaction, B2 converted 98.9% of albiflorin, which was better than the 24.5% conversion observed under the reaction conditions of the Tris-HCl buffer solution, and produced 57.6 μg/ml of benzoic acid in PBS buffer, which was better than the 13.6 μg/ml produced in Tris-HCl buffer. B3 converted 35.2% of the albiflorin in PBS buffer, which is slightly lower than the 36.1% converted in Tris-HCl buffer, and the production of benzoic acid in PBS buffer was also slightly lower than that in Tris-HCl buffer. As shown in Figures 4C,D, the amount of albiflorin converted by B4 was reduced by 46.7% in Tris-HCl buffer, which was better than the 20.6% conversion rate in PBS buffer. The amount of albiflorin converted by BL was reduced by 54.5% in Tris-HCl buffer, which was better than 20.6% conversion rate in PBS buffer. However, under the two buffer conditions, B4 and BL produced very little benzoic acid.

Finally, the effect of different pH buffer reaction conditions on the conversion of albiflorin by the four enzymes was investigated. As shown in Figure 4E, under the conditions of pH 6.0, pH 7.0, and pH 8.0, after 24 h of reaction, B2 converted 98.4, 96.6, and 97.8% albiflorin, respectively, and B3 converted 23.7, 44.0, and 0% albiflorin, respectively. As shown in Figure 4F, under the conditions of pH 6.0, pH 7.0, and pH 8.0, after 24 h of reaction, B2 produced 91.2, 88.8, and 93.8 μg/ml benzoic acid, respectively, and B3 produced 24.6, 21.2, and 1.6 μg/ml benzoic acid, respectively. As shown in Figure 4G, at pH 6.0 and pH 7.0, after 24 h of reaction, B4 converted 21.1 and 20.6% albiflorin, respectively, but the conversion at pH 8.0 was not obvious. The conversion rate of albiflorin by BL was 15.4 and 11.5% at pH 6.0 and pH 8.0, respectively, and little conversion occurred at pH 7.0. As shown in Figure 4H, after 24 h at pH 6.0, pH 7.0, and pH 8.0, B4 and BL produced very little benzoic acid.

### Bacterial Transplantation Affects Drug Metabolism of Albiflorin *in vivo*

**Figure 5:**
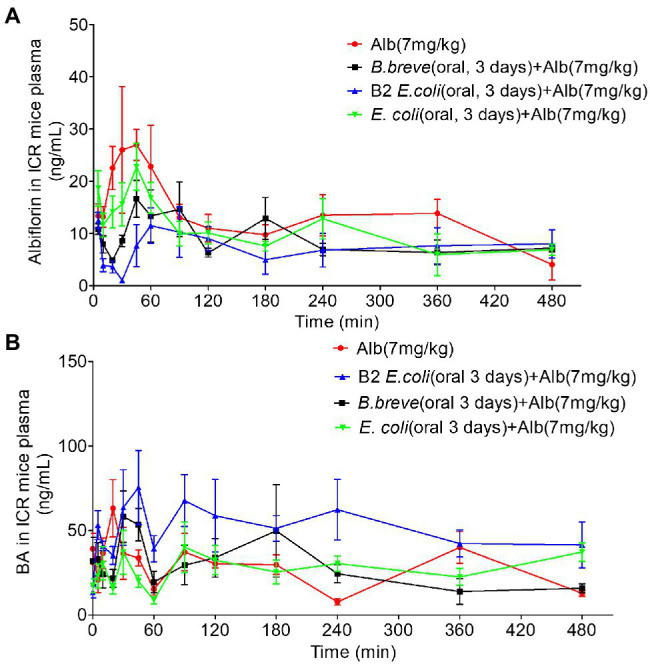
Pharmacokinetic study of albiflorin *in vivo*. **(A)** Plasma concentration-time profiles of albiflorin (Alb) in four groups of mice (oral 3 days, NS, *Bifidobacterium breve* 1 × 10^10^ CFU/day, *Escherichia coli* with B2 1 × 10^10^ CFU/day, and *E. coli* 1 × 10^10^ CFU/day) after oral administration (7 mg/kg). **(B)** Plasma concentration-time profiles of Benzoic acid (BA) in four groups of mice (oral 3 days, NS, *B. breve* 1 × 10^10^ CFU/day, *E. coli* with B2 1 × 10^10^ CFU/day, and *E. coli* 1 × 10^10^ CFU/day) after oral administration of albiflorin (7 mg/kg).

After 3 days of oral administration of different bacteria (Normal saline, NS, *E. coli* 1 × 10^10^ CFU/day, *E. coli* with Esterase B2 1 × 10^10^ CFU/day, *B. breve* 1 × 10^10^ CFU/day), ICR mice were given a single dose of albiflorin (7 mg/kg) to investigate the differences in albiflorin and benzoic acid concentrations over time in the four groups (Figures 5A,B). Pharmacokinetic parameters were shown in Supplementary Table S3. The AUC_(0– t)_ of the four groups were 5,965.590 ± 2,004.089, 5,452.317 ± 1,045.409, 4,254.94 ± 1,591.654, and 4,355.606 ± 934.475 μg/L h, respectively. The *C*_max_ of the four groups were 44.064 ± 24.12, 45 ± 10.607, 21.61 ± 5.363, and 26.528 ± 11.105 μg/L, respectively. Similar pharmacokinetic parameters were observed in the NS group and the *E. coli* group. The transplantation of *E. coli* had little effect on the drug metabolism of albiflorin. The administration of *E. coli* containing esterase B2 obviously affected the absorption and metabolism of albiflorin *in vivo*, while the administration of *B. brevis* containing esterase B2 also affected the metabolism of albiflorin *in vivo* to a certain extent. At the same time, the plasma concentration-time profiles of albiflorin metabolites benzoic acid were determined, the results showed that the benzoic acid concentration of B2 group obviously greater than the NS group, the *B. breve* group could also increase the benzoic acid concentration in plasma, and the benzoic acid concentration in plasma of *E. coli* group and NS group is similar. *In vivo* experiments showed that supplement of esterase B2 could affect the metabolism of albiflorin in mice and significantly increased the concentration of benzoic acid, the metabolite of albiflorin in plasma. Esterase B2 is an important enzyme for the conversion of albiflorin to benzoic acid.

## Discussion

As a result of the in-depth study of gut microbiota, increasing evidence shows that gut microbiota is involved in drug metabolism, which plays a key role in determining the therapeutic effect and host metabolism (Sousa et al., 2009; Spanogiannopoulos et al., 2016; Koppel et al., 2017; Wu and Tan, 2018). Different bacteria produce different metabolic enzymes, such as β-glucuronidase, β-galactosidase, β-glucosidase, nitroreductase, azo reductase, protease, 7-αhydroxylase, and various carbohydrate enzymes, which are responsible for metabolizing different drugs (Schackmann, 1992; Lavrijsen et al., 1995; Ionescu and Caira, 2005; Pollet et al., 2017). Many natural compounds have low bioavailability, and their metabolism depends on the gut microbiota. Among them, natural compounds containing ester bonds are very abundant, including chlorogenic acid, geniposide, asiaticoside, ferulic acid, caffeic acid, and motherwort. Our previous laboratory research showed that albiflorin can be metabolized by intestinal bacteria (Zhao et al., 2018). By comparing the ability of 18 intestinal bacterial strains, we found that four bacteria had a significant ability to metabolize albiflorin, including *B. breve*. *Bifidobacteria* is an important constituent of human intestinal bacteria (accounting for 2–10%), and it contains abundant enzyme resources, such as α-glucosidase, β-glucosidase, hydrolase, and esterase (Kim and Lee, 2010; Kim et al., 2017; Florindo et al., 2018).

Several taxa at the family and genus levels, specifically family Prevotellaceae, genus *Corprococcus*, and *Faecalibacterium*, were decreased in major depressive disorder (MDD) compared to non-depressed controls in observational studies, and depressive symptoms were improved compared to controls in interventional studies with probiotics (Sanada et al., 2020). Studies have shown that *B. longum* and *B. breve* strains reduce stress-, anxiety-, and depression-related behaviors in male BALB/c mice with congenital anxiety. This beneficial effect is related to its influence on enteric neurons and vagus nerve signals (Savignac et al., 2014). Preclinical and clinical studies have shown that *Bifidobacteria* may have therapeutic effects on mood disorders (Desbonnet et al., 2010). In addition to its beneficial effect on human health, *Bifidobacteria* was also shown to affect TCM efficacy. For example, daidzin, a glycosidic isoflavone that mainly exists in soy products, can be metabolized into equol by *Bifidobacterium via* glycosidic cleavage and reduction of an α, β-unsaturated ketone (Setchell and Clerici, 2010). For *Bif. animalis* subsp. *lactis*, the metabolism of caffeic acid and chlorogenic acid was described (Couteau et al., 2001; Raimondi et al., 2015; Fritsch et al., 2016a).

In this study, we used four common *Bifidobacteria* strains from the intestinal flora to examine the metabolic effect of *Bifidobacteria* on albiflorin. Albiflorin can be converted into benzoic acid, which is the main metabolite, by intestinal bacteria in large amounts. This is similar to the antidepressant mechanism of albiflorin speculated previously. According to the comparison of the conversion abilities of the four *Bifidobacteria* strains and genome mining results, we selected *B. breve* and *B. longum*, which have a strong albiflorin metabolism ability. Previous studies have shown that esterases from *B. animalis* subsp. *lactis* DSM 10140 and *B. animalis* subsp. *lactis* WC 0432 were able to hydrolyze HCA-containing substrates and chlorogenic acid (Raimondi et al., 2015; Fritsch et al., 2016b). In this study, three esterases, B2, B3, and B4, from *B. breve* and one esterase, BL, from *B. longum*, which belong to three superfamilies, were identified and characterized. Esterase (EC 3.1.1.1), which is generally known as carboxylesterase, belongs to the class of hydrolytic enzymes, the function of which is to hydrolyze carboxylic acid esters (Porro et al., 2019). It can catalyze ester bond cleavage and form the corresponding alcohols and acids in the presence of H_2_O molecules. By using SWISS-MODEL, it was found that all four esterases have the core domain of esterase, but the surrounding regions are very different. These enzymes are composed of highly similar α/β-hydrolase folds and different helix domains. The catalytic apparatus of alpha/beta hydrolases typically involves three residues (catalytic triad): a serine, a glutamate/aspartate, and a histidine. The catalytic mechanism often involves a nucleophilic attack on a carbonyl carbon atom (Jochens et al., 2011; Bauer et al., 2020).

Then, the albiflorin metabolism function of the four esterases was verified. It was demonstrated that at 37°C, B2 and B3 showed stronger hydrolysis function and produced more benzoic acid. B4 had a weaker metabolic capacity, and BL could not convert albiflorin. To explore the influence of the enzyme reaction conditions, different temperatures, buffers, and pH conditions were investigated to explore the most suitable reaction conditions for the four enzymes. It is revealed that at 37°C, PBS buffer and pH 7.0 are the optimal conditions for B2, which has the strongest ability to convert albiflorin. This result indicated that B2 might be the most important esterase and play a crucial role in hydrolysis by *B. breve* to convert albiflorin. Furthermore, by pharmacokinetic analysis of albiflorin in animals, we verified that transplantation of *E. coli* containing esterase B2 affected the drug metabolism of albiflorin, and the effect was more obvious than that of *B. brevis* containing B2. Recent studies by Zimmermann et al. (2019a,b) have revealed that the contributions of the microbiome to the metabolism of some drugs are much more than 50%, it provided insight into the important roles of the gut microbiota in the metabolism of many pharmaceuticals. For instance, some phenolic compounds with low bioavailability, such as rosmarinic acid and eriodictyol, are fermented into absorbable and bioactive phenolic acids by the gut microbiota, e.g., hydroxyphenylpropionic acids and phenylpropionic acids (Jochens et al., 2011; Zimmermann et al., 2019a). These bioactive microbial metabolites may be absorbed and transported by the circulatory system to tissues and organs or exert their effects in the intestinal lumen (Aura, 2008; Hanske et al., 2009; Mosele et al., 2014). Gut bacteria produce a range of enzymes that might chemically alter drugs as varied as psychotropics and cancer treatments, rendering them less useful or leading to more side effects (Paolo et al., 2018; Spanogiannopoulos and Turnbaugh, 2018).

In summary, this study examined the metabolism of albiflorin, a compound with antidepressant activity, by the enzymes contained in intestinal bacteria and confirmed that benzoic acid is the main metabolite. The conversion of albiflorin to benzoic acid through an *in vitro* reaction by rat intestinal bacteria was verified, and the four esterases in *B. breve* and *B. longum* that play an important role in the metabolism of natural compounds were identified through a bioinformatics search. Furthermore, their albiflorin metabolism functions were compared, and the best enzyme reaction conditions were verified. This study revealed the mechanism by which *Bifidobacteria* convert natural compounds containing ester bonds and characterized their specific functional enzymes. TCM herbs closely interact with gut microbiota and affect their composition (Peng et al., 2020). Reciprocally, the gut microbiota also plays essential roles in the conversion of carbohydrates, proteins, lipids, and non-nutritive small chemical compounds from TCM herbs into chemical metabolites that may show beneficial or adverse effects on human health (Feng et al., 2019; Yue et al., 2019). This discovery presents new prospects for the development of new enzymes and probiotics that can be used to enhance the bioconversion of TCM chemicals into bioactive compounds and reveals a new way for *Bifidobacteria* to assist in the treatment of depression and other mental diseases. However, the discovery, development, and functional characterization of *Bifidobacteria* esterases still need more research. A deeper understanding of the mechanism of the drug-gut microbiota interaction is required to guide the research and development of diet or drug interventions targeting the microbiome, which may have the potential to enhance drug efficacy or reduce adverse drug reactions. Understanding the interplay between microbes and medicine could lead to new therapies, or to changes in how existing drugs are prescribed.

## Data Availability Statement

The original contributions presented in the study are included in the article/Supplementary Material, further inquiries can be directed to the corresponding author.

## Ethics Statement

This study was performed in accordance with the recommendations of guidelines for Animal Experimental Center, Animal Care and Welfare Committee, Institute of Materia Medica, CAMS and PUMC. The protocol was approved by the Animal Care and Welfare Committee, Institute of Materia Medica, CAMS PUMC.

## Author Contributions

YW conceptualized the experiments and analyses and project administration. RP performed the molecular biology study. RP, PH, Z-WZ, and JF performed the bioconversion study. L-BP, HY, and HX analyzed the data. YW, RP, and S-RM contributed to the writing-review and editing. Y-YX and C-XL contributed to the revision of the manuscript. All authors contributed to the article and approved the submitted version.

## Funding

The project was supported by CAMS Innovation Fund for Medical Sciences (CIFMS) (nos. 2016-I2M-3-011, 2019-I2M-5-020, and 2021-1-I2M-007) the National Natural Science Foundation of China (nos. 81973290 and 82173888), Beijing Natural Sciences Fund Key Projects (no. 7181007), the Beijing Key Laboratory of Non-Clinical Drug Metabolism and PK/PD study (Z141102004414062), and the Youth Science Foundation Project from National Natural Science Foundation of China (no. 81803613).

## Conflict of Interest

The authors declare that the research was conducted in the absence of any commercial or financial relationships that could be construed as a potential conflict of interest.

## Publisher’s Note

All claims expressed in this article are solely those of the authors and do not necessarily represent those of their affiliated organizations, or those of the publisher, the editors and the reviewers. Any product that may be evaluated in this article, or claim that may be made by its manufacturer, is not guaranteed or endorsed by the publisher.

## Footnotes

^1^ http://www.ncbi.nlm.nih.gov

^2^ https://swissmodel.expasy.org

^3^ https://web.expasy.org/protparam/
