# Supplementary material for: Esterases From Bifidobacteria Exhibit the Conversion of Albiflorin in Gut Microbiota
Source: Front Microbiol. 2022 Apr 6;13:880118. doi: 10.3389/fmicb.2022.880118 (PMC9019491; doi:10.3389/fmicb.2022.880118)
Supplement: Supplementary file 1 [file Table_1.DOCX]

**Supplemental Information**

**Table S1.** Strains and plasmids used in this study.

| **Strain and plasmid** | | | **Relevant characteristics** | **Source or reference** | |
| --- | --- | --- | --- | --- | --- |
| **Strain** | |  | | |  |
| ***E. coli*** | | |  | |  |
| DH5α | | | F^−^, *ΔlacU169 (ϕ80lacZΔM15), hsdR17, supE44, recA1, gyrA96, endA1, relA1，thi‑1* | | Life Technologies |
| BL21 (DE3) | | |  | | Life Technologies |
| ***Bifidobacterium*** | | |  | |  |
| *Bifidobacterium breve* ATCC15700 | | | Type strain | | ATCC |
| *Bifidobacterium longum* ATCC15697 | | | Type strain of *Bifidobacterium infantis* | | ATCC |
| *Bifidobacterium animalis* ATCC27673 | | | Wild type | | ATCC |
| *Bifidobacterium adolescentis* ATCC15703 | | | Type strain | | ATCC |
| **Plasmid** | | |  | |  |
| pET-28a | | | Expression vector | | Novagen |
| pET-28a-*b2* | | | Ligating the *b2* gene to the pET-28a | | This study |
| pET-28a- *b3* | | Ligating the *b3* gene to the pET-28a | | This study |  |
| pET-28a- *b4* | | Ligating the *b4* gene to the pET-28a | | This study |  |
| pET-28a- *bl* | | Ligating the *bl* gene to the pET-28a | | This study |  |

**Table S2.** List of primers involved in this study.

| Primers | Sequences (5' to 3') |
| --- | --- |
| b2-F | agcaaatgggtcgcggatccATGGCACAGCCGCAGCCGTATTAC |
| b2-R | tcgagtgcggccgcaagcttTCATCTCAACGGAGCGAACGCCATA |
| b3-F | agcaaatgggtcgcggatccATGGCAATAGAACTGGCCAATCAT |
| b3-R | tcgagtgcggccgcaagcttTCAGTGTTGTTGCACGCGCTTG |
| b4-F | agcaaatgggtcgcggatccGTGAGCGGGGCGGGGCGCATAC |
| b4-R | tcgagtgcggccgcaagcttTTATATTTCGCCGGAAACATGAG |
| bl-F | agcaaatgggtcgcggatccATGAGCAAAGAAGCGAATGCCAG |
| bl-R | tcgagtgcggccgcaagcttCTACAGCACGCCAGAAGTAATGTCG |
| pET28-F | AAGCTTGCGGCCGCACTC |
| pET28-R | GGATCCGCGACCCATTTG |

**Table S3.** Pharmacokinetic parameters of mice after microbiota transplantation(oral 3 days, then oral 7mg/kg albiflorin) in this study.

| Pharmacokinetic parameters | NS  (Oral normal saline+7mg/kg Abl) | *E. coli*  （Oral *E.coli*+7mg/kg Abl) | B2  （Oral *E.coli* with B2+7mg/kg Abl)) | *B. breve*  （Oral *B. breve*+7mg/kg Abl) |
| --- | --- | --- | --- | --- |
| AUC_(0-t)_ (μg/L*h) | 5965.590±2004.089 | 5452.317±1045.409 | 4254.94±1591.654 | 4355.606±934.475 |
| AUC_(0-∞)_ (μg/L*h) | 7700.423  ±3138.343 | 10312.98±5934.235 | 5879±2859.299 | 6641.5836±3539.551 |
| MRT_(0-t)_ (h) | 187.635±42.751 | 246.22±216.314 | 255.414±79.36 | 222.811±18.559 |
| MRT_(0-∞)_ (h) | 359.447±203.024 | 509.159±237.899 | 398.481±164.506 | 502.194±325.752 |
| t_1/2z_ (min) | 223.802±165.446 | 145.325±81.007 | 154.295±128.802 | 182.516±77.505 |
| T_max_ (min) | 40.833±13.934 | 45±10.607 | 81±29.240 | 93.333±72.503 |
| V_z_(L/kg) | 311.649±280.346 | 146.45±69.436 | 227.298±137.166 | 341.148±135.832 |
| CL_z_(L/min/kg) | 0.896±0.618 | 0.831±0.54 | 1.584±1.022 | 1.144±0.608 |
| C_max_ (μg/L) | 44.064±24.12 | 45±10.607 | 21.61±5.363 | 26.528±11.105 |
